# Gemella sanguinis: A Rare Cause of Endocarditis in a Bicuspid Aortic Valve

**DOI:** 10.7759/cureus.78837

**Published:** 2025-02-11

**Authors:** Emily L King, Ashton R McDonald, Abdul Muhsen Z Abdeen, Davinder Singh, Christine Gilkerson

**Affiliations:** 1 Internal Medicine, Marshall University Joan C. Edwards School of Medicine, Huntington, USA

**Keywords:** bicuspid aortic valve disease, gemella, gemella endocarditis, gemella sanguinis, infective endocarditis

## Abstract

Here, we present a case of infective endocarditis (IE) caused by *Gemella sanguinis, *a pathogen that rarely causes human infection. The patient in this case was an otherwise healthy 50-year-old man who presented with gradually worsening shortness of breath, weight loss, fatigue, chills, and leg swelling for six weeks. He had no prior history of cardiac disease and had never used intravenous drugs, but he had undergone extensive dental work following a military-associated injury. A workup for IE was done, and echocardiography revealed a bicuspid aortic valve (BAV) with vegetations causing moderate to severe aortic regurgitation. Blood cultures, which initially showed only gram-positive cocci, later identified *Gemella sanguinis*. He subsequently recovered after receiving treatment with antibiotics and undergoing an aortic valve replacement. The patient’s BAV and significant dental history acted as risk factors for this condition, and although his symptoms were nonspecific and he did not exhibit other classic risk factors for IE, his case represents the importance of keeping high clinical suspicion to allow for prompt initiation of appropriate treatment. More research on *Gemella* is needed, as these species are difficult to identify and therefore may be a more significant cause of infection than is currently known.

## Introduction

Infective endocarditis (IE) is a relatively rare condition, with an annual incidence of three to 10 cases per 100,000 people [1]. Despite improvements in diagnostics and treatments, IE has continued to have high rates of mortality over the past four decades, with estimated rates of in-hospital mortality around 20% and one-year mortality exceeding 30% [2]. IE also carries a heavy financial burden, as the inflation-adjusted expenditure on IE hospitalizations in the United States increased from $1.56 billion in 2003 to $2.34 billion in 2016. In recent decades, advancements in medicine have altered the clinical presentation and populations most at risk for IE. Rheumatic heart disease, which was previously the most common predisposing condition worldwide, has substantially decreased in developed countries, while structural heart conditions, such as bicuspid aortic valve (BAV), and intracardiac devices have increased to become the most common risk factors for IE in developed countries [3]. Also, an aging population and increasing numbers of comorbidities, nosocomial exposures, prosthetic valves, intravenous drug use (IVDU), and patients undergoing hemodialysis have changed the populations most likely to be affected by IE. Around 80% to 90% of all IE cases are caused by streptococci, staphylococci, and enterococci, with *Staphylococcus aureus* responsible for roughly 30% of cases in the developed world [4]. Diagnosing IE can be challenging, as patients often present atypically and may not always have evident risk factors.

*Gemella sanguinis* is a catalase-negative, facultative anaerobic, gram-positive coccus that shows hemolytic activity and normally colonizes the human oral cavity and gastrointestinal tract [5]. Although *Gemella* are usually commensal organisms, seven species are known to cause human infection, and they most often affect the cardiac valves [6,7]. However, such infections are extremely rare, and *G. sanguinis *is the least common among them [8]. Because *Gemella* are easily decolorized during gram staining, they can be challenging to identify and may be responsible for more infections than is currently recognized.

## Case presentation

A 50-year-old male originally presented to his primary care provider’s office with complaints of gradually worsening dyspnea on exertion, unintentional weight loss of 15 pounds, fatigue, night sweats, chills, and bilateral leg swelling for six weeks. He had no significant medical history; however, he reported undergoing extensive dental work over the span of many years after he fell from a building at 19 years old while on a military deployment. The fall caused significant dental trauma, resulting in multiple crowns, implants with bovine grafts, and dentures for his upper teeth, which were placed around age 40. He had not undergone any recent dental procedures and had not visited a dentist in several years. He denied any history of or current IVDU. A physical examination displayed a blood pressure of 130/80, a pulse of 93, and an oxygen saturation of 100%. The patient’s primary care physician ordered laboratory tests, which revealed anemia and elevated brain natriuretic peptide (BNP), and he was subsequently referred to cardiology, gastroenterology, and hematology/oncology for further evaluation.

**Figure 1:**
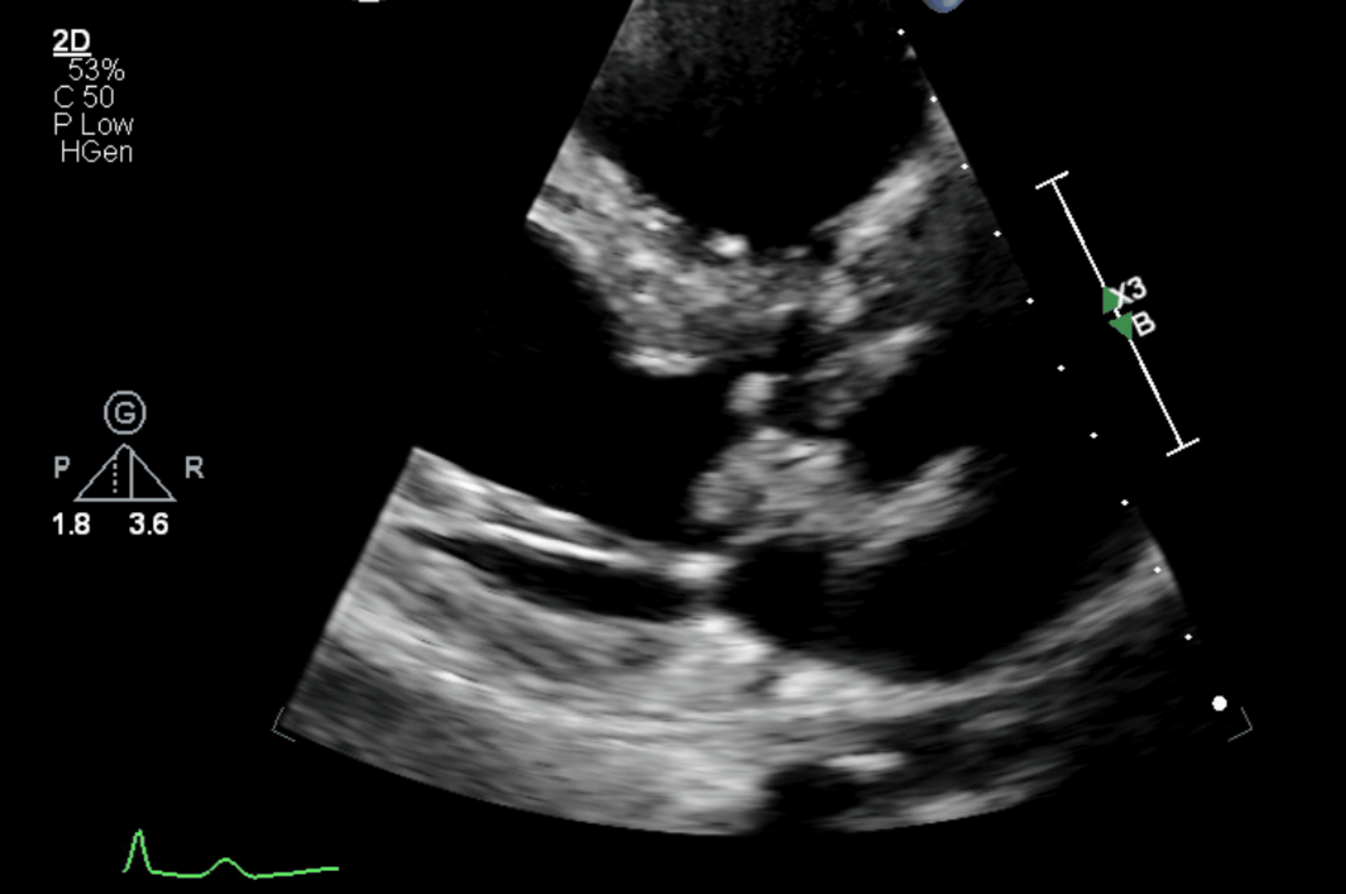
Transthoracic echocardiography photo of a large vegetation on the minor cusp of the aortic valve.

**Figure 2:**
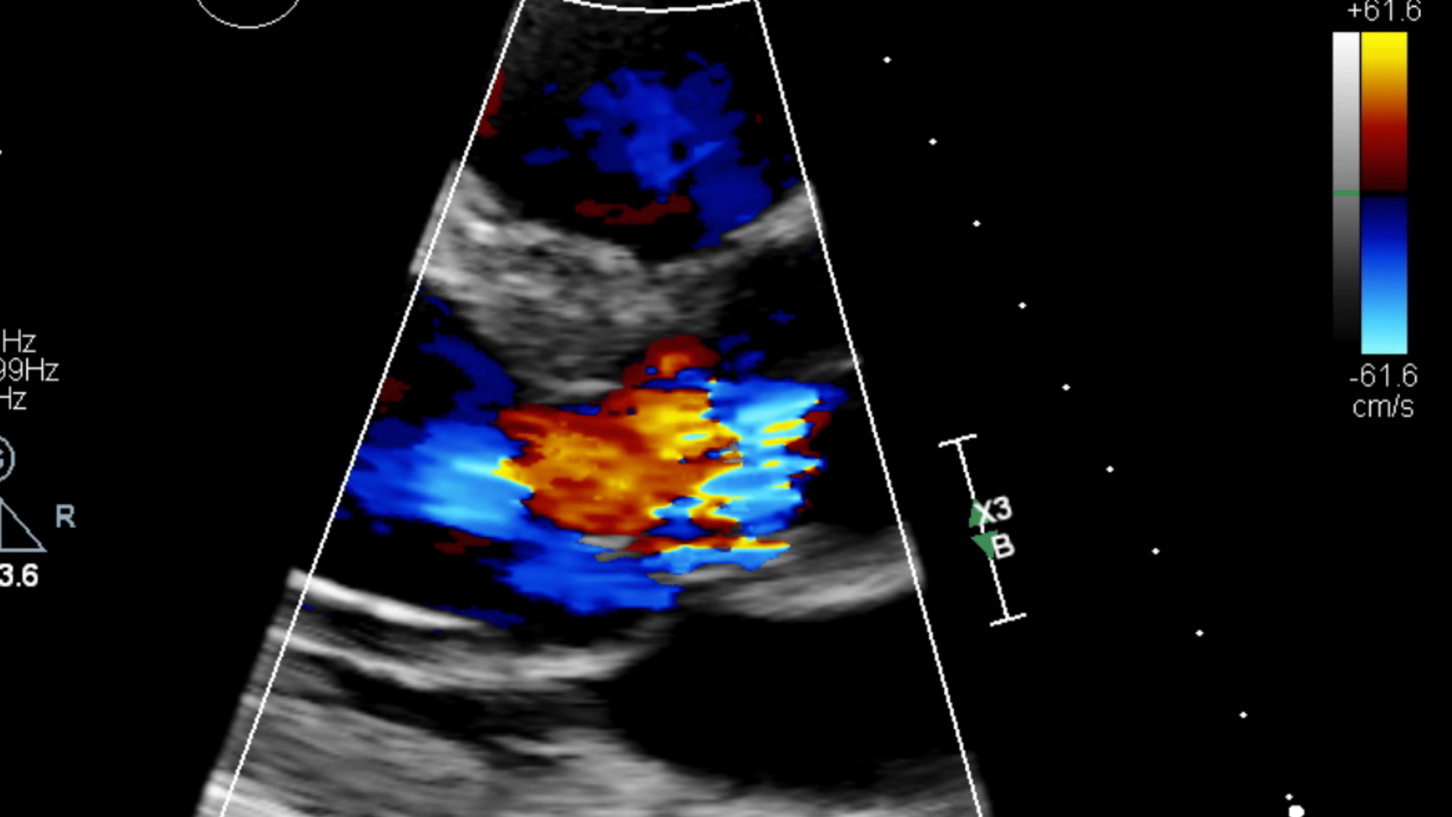
Moderate to severe aortic regurgitation seen on transthoracic echocardiography.

**Figure 3:**
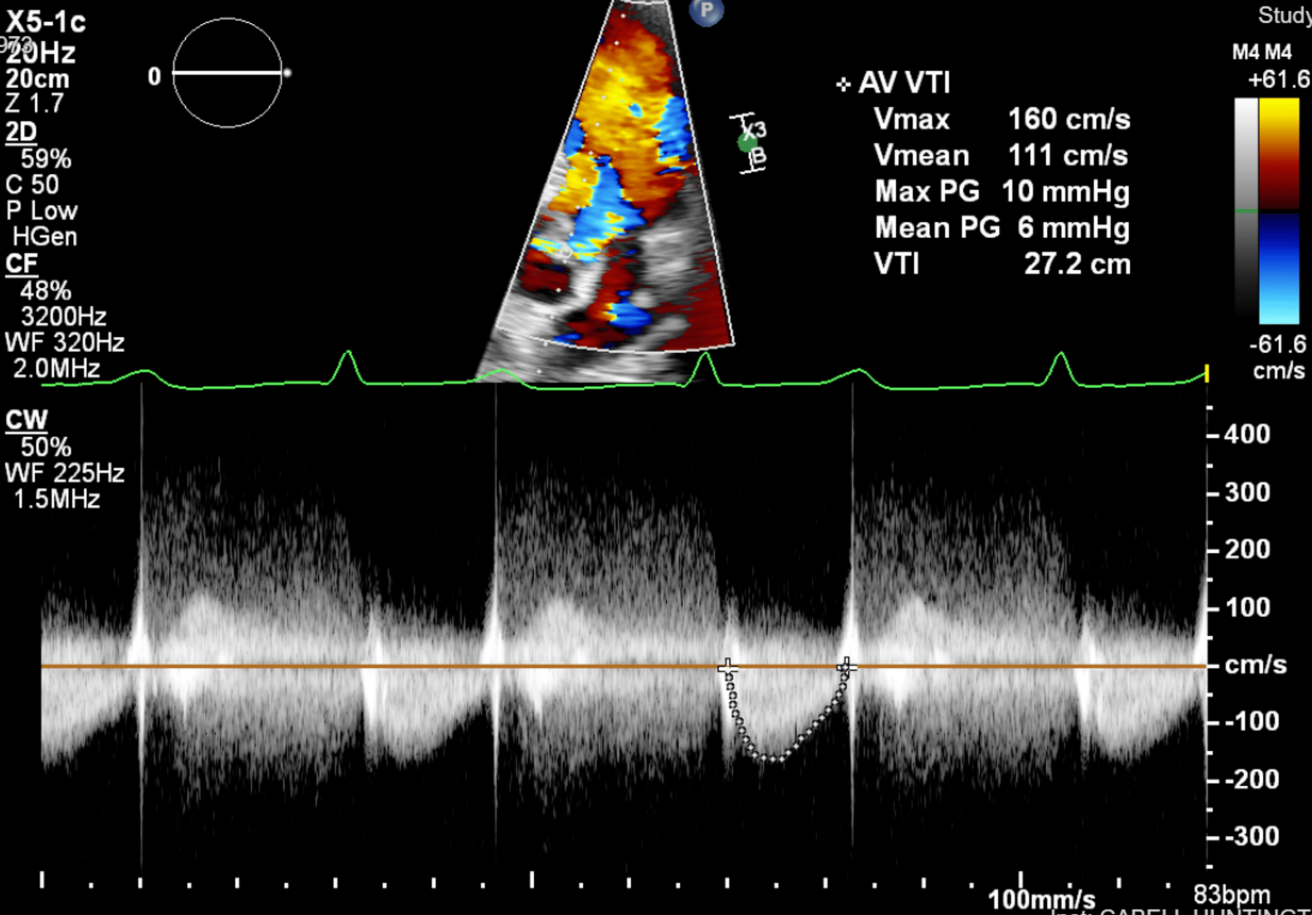
Transthoracic echocardiography Doppler showing aortic regurgitation.

Three days later, the patient presented to a local hospital’s emergency department (ED) due to worsening pain in his right leg. He also reported a fever of 103°F the previous night, though he was afebrile at the time of ED evaluation. Imaging performed included an unremarkable chest X-ray, Doppler ultrasound, and CT of the right leg without evidence of deep vein thrombosis, and an abdominal CT showing splenomegaly and left renal calculi with no ureteral calculus or obstructive change. Urinalysis was notable for mild hematuria. Blood cultures were drawn and he was discharged home from the ED. The next day, the patient was instructed to return to the ED after blood cultures revealed an unidentified genus of gram-positive cocci. The patient was admitted to the hospital and was started on empiric intravenous (IV) vancomycin and piperacillin-tazobactam. Transthoracic echocardiography (TTE) identified a BAV with a large vegetation measuring 1.8 × 1.1 cm, which resulted in moderate to severe aortic insufficiency (Figures 1-3). Upon admission, the patient had a fever of 102.9°F. Five repeat blood cultures over the span of two days were taken at this facility, which initially showed gram-positive cocci, but were later identified as *Gemella sanguinis*.

The patient was transferred to another hospital for specialized management of IE, and consultations with cardiology, infectious disease, and cardiovascular and thoracic surgery (CVT) were obtained. Empiric IV antibiotic therapy was continued until repeat blood cultures were taken, which again were positive for *G. sanguinis*. However, antibiotic susceptibility testing was not performed as the blood culture samples were unsuitable for testing. The empiric treatment of piperacillin-tazobactam and vancomycin was discontinued, and IV ceftriaxone was started. Though penicillin G would be the preferred choice, minimal inhibitory concentration (MIC) for penicillin was unable to be determined, and ceftriaxone has similar efficacy to penicillin in treating many gram-positive organisms while providing narrower, more specific coverage than the empiric regimen. Repeat blood cultures later on the day of transfer showed no growth.

Physical examination showed 2+ pitting edema to the bilateral lower extremities with no skin discoloration, warmth, or tenderness. The cardiac examination was normal, and no murmurs were appreciated. At the time of this evaluation, he was febrile with a temperature of 101.3°F and hypertensive with a blood pressure of 158/100 mmHg. His other vital signs were within normal limits, and the rest of his physical exam was unremarkable.

Laboratory investigations at the new facility (Table 1) revealed hemoglobin at 8.9 g/dL, hematocrit at 27.6%, mean corpuscular volume (MCV) of 89.9 fL, platelets count of 92,000/mcL, erythrocyte sedimentation rate (ESR) of 95 mm/hr, total protein at 3.0 g/dL, calcium at 7.1 mg/dL (ionized calcium: 1.16 mmol/L), and glucose at 158 mg/dL. The white blood cell count was 6,300/μL, which was within the normal range. Other parameters, including electrolytes, blood urea nitrogen, creatinine, liver enzymes, and lactic acid, were unremarkable.

**Table 1:** Selection of patient's labs at admission with normal reference ranges.

Lab parameter	Patient’s values at admission	Normal reference ranges (for adult males)
Hemoglobin	8.9 g/dL	13.5–17.5 g/dL
Mean corpuscular volume (MCV)	89.9 fL	80–100 fL
Hematocrit	27.6%	41.0–50.0%
Platelet count	92,000/mcL	150,000–450,000/mcL
Erythrocyte sedimentation rate (ESR)	95 mm/hr	<20 mm/hr
Total protein	3.0 g/dL	6.0–8.3 g/dL
Calcium (total)	7.1 mg/dL	8.5–10.5 mg/dL
Calcium (ionized)	1.16 mmol/L	1.12–1.32 mmol/L
Glucose	158 mg/dL	<140 mg/dL (non-fasting)
White blood cell count (WBC)	6,300/μL	4,000–11,000/μL

**Figure 4:**
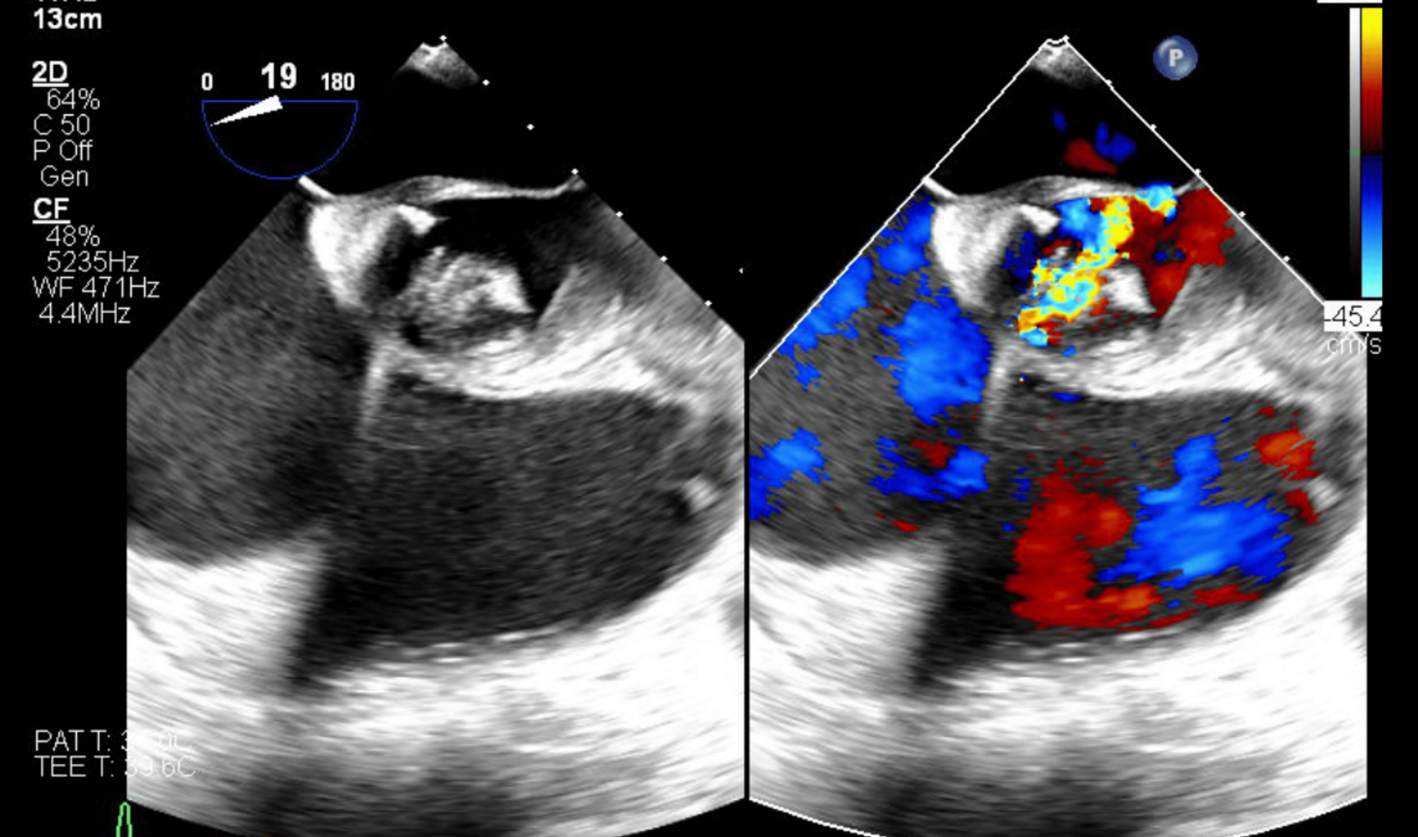
Photos of transesophageal echocardiography showing vegetation on the left and regurgitation on the right.

Further workup included a panorex dental X-ray, which was performed to look for a potential source of infection from the oral cavity, showing no acute findings, and left heart catheterization showing normal coronary arteries and ejection fraction. Results of transesophageal echocardiography (TEE) revealed a small papillary mobile vegetation on the noncoronary cusp and a large mobile pedunculated vegetation measuring 2.2 x 0.6 cm in the subvalvular apparatus of the aortic valve along with moderate to severe aortic regurgitation (Figure 4). The patient underwent aortic valve replacement (AVR) with a tissue valve without complications. A peripherally inserted central catheter (PICC) line was placed one day prior to discharge through which he continued to receive IV ceftriaxone for four weeks total. Since his discharge, he has remained stable, has followed up with the infectious disease and CVT teams, and has participated in cardiac rehabilitation.

## Discussion

**Figure 5:**
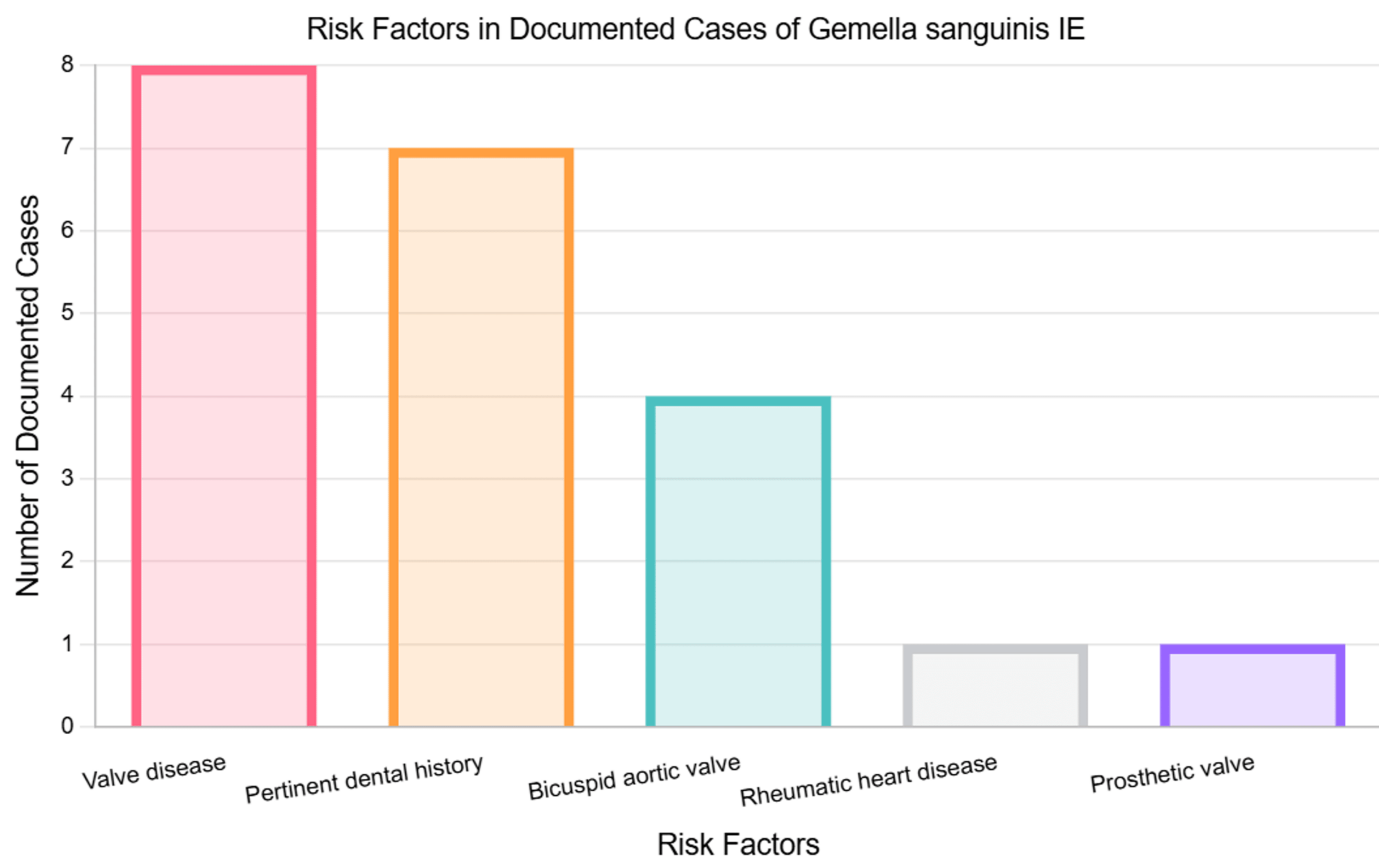
Risk factors in documented cases of Gemella sanguinis infective endocarditis (IE).

*Gemella sanguinis* is a gram-positive coccus that normally exists as a commensal organism in humans, but can occasionally become an opportunistic pathogen that particularly affects cardiac valves [7]. *G. sanguinis*-associated IE is rare, with only 16 published cases in the medical literature as of 2025, as shown in Table 2 [5-20]. Out of these reported cases, eight had a history of valve disease, with four of these having BAV. Also, seven had pertinent past histories of oral involvement, such as recent dental infections or procedures. The number of cases with these risk factors is demonstrated in Figure 5. In the case reported here, the patient had a BAV of which he was previously unaware, along with a 30-year history of extensive dental work, including bovine implants and dentures.

**Table 2:** Summary of published cases along with the present reported case of Gemella sanguinis-associated infective endocarditis.

Case	Year	Reference number	Age/gender	Cardiac risk factors	Oral cavity involvement	Valve(s) involved	Vegetation present?	Antibiotic regimen	Valve replacement?	Outcome
Collins et al.	1998	[9]	69 M	Unknown	Unknown	Unknown	Unknown	Unknown	Unknown	Unknown
Shukla et al.	2002	[10]	69 M	No	Periodontal disease, tooth abscess	Mitral	Yes	Vancomycin + gentamicin	Yes	Recovery
Lee et al.	2009	[11]	73 M	Unknown	Dental infection	Mitral	Yes	Ampicillin-sulbactam + gentamicin	No	Death
Almaghrabi et al.	2009	[12]	23 M	Repaired ventricular septal defect, aortic valve regurgitation	Behçet disease (mouth ulcers)	Aortic	Yes	Ceftriaxone + gentamicin	No	Death
Tiu et al.	2012	[13]	27 F	Rheumatic heart disease, aortic/mitral valve replacement	Dental infection, recent root canal	Prosthetic aortic	No	Ceftriaxone	Yes	Recovery
Yang et al.	2012	[14]	67 M	Bicuspid aortic valve	None	Aortic	No	Penicillin G	Yes	Recovery
Chadha et al.	2013	[15]	73 M	None	None	Aortic, mitral	Yes	Daptomycin + gentamicin	Yes	Recovery
Rousseau-Gagnon et al.	2013	[16]	67 M	None	Dental infection	Aortic, mitral, tricuspid	Yes	Penicillin G + gentamicin	Yes	Recovery
Tsumita et al.	2015	[17]	57 F	None	None	Aortic	Yes	Vancomycin + gentamicin	Yes	Recovery
Mugunthan et al.	2016	[18]	4 M	None	None	Tricuspid	Yes	Vancomycin + gentamicin	Yes	Recovery
Maraki et al.	2019	[5]	21 M	Bicuspid aortic valve	Recent dental cleaning, molar extraction	Aortic	Yes	Ceftriaxone + gentamicin	Yes	Recovery
Emmanouilidou et al.	2019	[19]	85 F	Aortic regurgitation, mitral insufficiency	None	Mitral	No	Vancomycin + gentamicin	No	Recovery
Sideris et al.	2020	[6]	53 M	Mitral valve prolapse	Recent root canal procedure	Mitral	Yes	Penicillin + ceftriaxone	Yes	Recovery
Shah et al.	2022	[7]	53 M	None	None	Aortic, mitral	Yes	Vancomycin, cefepime + doxycycline	Yes	Death
Filip et al.	2023	[8]	8 M	Shone's syndrome with a bicuspid valve, mitral parachute valve, and severe aortic coarctation	None	Aortic	Yes	Meropenem + linezolid	Yes	Recovery
Amendolagine et al.	2023	[20]	52 F	Bicuspid aortic valve	None	Aortic	Yes	Vancomycin + ceftriaxone	Yes	Recovery
Present case	2024	-	50 M	Bicuspid aortic valve	Extensive dental history (implants, dentures) but no recent procedures	Aortic	Yes	Ceftriaxone	Yes	Recovery

Patients with BAV develop IE at significantly higher rates than the general population, with a reported 10% to 30% of patients with BAV developing IE [21]. Also, 25% of all IE cases develop in patients with BAV. Individuals with BAV often have IE only in the aortic valve, and the different flow patterns through the abnormal valve could explain the endothelial damage that permits bacterial adhesion and deposition of platelets and fibrinogen, leading to vegetation formation [22]. Much about how *Gemella* causes opportunistic invasive infections remains unknown [23]. There is some data to suggest the gene pool of the oral bacterial environment permits *Gemella* to survive and cause invasive diseases such as endocarditis or sepsis.

TTE is commonly used as the first-line imaging modality for the diagnosis of IE as it is non-invasive. In this case, initial TTE identified a 1.8 x 1.1 cm vegetation on the minor cusp of the aortic valve. TEE, which is more sensitive than TTE as it provides better visualization of posterior structures, showed a large mobile pedunculated vegetation measuring 2.2 x 0.6 cm in the subvalvular apparatus of the aortic valve in addition to another smaller vegetation. The echocardiograms also demonstrated the presence of moderate to severe aortic regurgitation, which was measured using Doppler parameters. According to the modified Duke criteria, the patient had definitive IE given that two major criteria were present: consecutive blood cultures positive for *G. sanguinis* obtained more than 12 hours apart and evidence of endocardial involvement found on TTE/TEE [24].

Treatment of IE generally requires prolonged courses of antibiotics chosen based on susceptibilities of the causative organisms. For empiric treatment, it is most important to cover staphylococcal, streptococcal, and enterococcal species as well as gram-negative bacilli [25]. *Gemella* isolates have been shown to be sensitive to beta-lactams and vancomycin [26]. In this case, piperacillin-tazobactam and vancomycin were started empirically before the organism was identified. The isolates were initially recognized as gram-positive cocci days before coming back specifically as *Gemella sanguinis*. *Gemella *are easily decolorized during gram staining, so it is likely they appeared as gram-positive cocci before additional testing could be performed. The next identification method used in the laboratory was matrix-assisted laser desorption/ionization-time of flight mass spectrometry (MALDI-ToF-MS) by Bruker (Billerica, MA), which was able to provide identification of the bacteria to the species. Unfortunately, antibiotic susceptibility testing was not performed as the blood culture samples were not suitable for testing. Since the penicillin minimum inhibitory concentration (MIC) was unavailable due to these limitations, the patient was switched to ceftriaxone, which provides coverage against *Gemella* species while avoiding unnecessary broad-spectrum activity against gram-negative and anaerobic organisms. The bacteremia was shown to be successfully treated as his repeat blood cultures detected no organism growth.

Valve replacement in patients with IE should not be delayed in suitable candidates to prevent progression to heart failure or other sequelae. The American Association for Thoracic Surgery (AATS) guidelines for the treatment of IE indicate surgery when patients with IE present with valve dysfunction resulting in symptoms of heart failure, have mobile vegetations exceeding 10 mm in length, or in cases of persistent bacteremia or fever that extend beyond five to seven days despite the initiation of suitable antimicrobial treatment [27]. In this patient’s case, surgical intervention was indicated as he had a large vegetation causing significant aortic regurgitation. The patient was informed of the risks and benefits of the procedure, and he elected to undergo tissue aortic valve replacement, which he recovered from without any significant complications such as bleeding, embolism, or infection. Out of the 16 previously reported cases, 12 reported recovery, with 11 of these patients receiving both antibiotic treatment and valve replacement.

IE can be classified as acute or subacute-chronic based on the timing of onset and severity of the clinical presentation. Given this patient’s subacute presentation and nonspecific symptoms of fatigue, weight loss, fever, and chills, there were many other diagnoses that could be on the initial differential. Some examples include pulmonary embolism, deep vein thrombosis, congestive heart failure, sepsis, autoimmune disorders, glomerulonephritis, or malignancies like colon cancer. At his initial emergency department visit, a chest X-ray did not reveal any pulmonary abnormalities, and CT and Doppler ultrasound of the right lower leg did not detect any thrombus. His labs did not show evidence of renal dysfunction, and his ejection fraction was normal on heart catheterization. Throughout his clinical course, other diagnostic tests such as urinalysis, lupus serology, and microbiology studies helped narrow the differential diagnosis and exclude other potential causes of the patient's symptoms. His labs also supported the diagnosis of IE, as anemia and hematuria are commonly seen in subacute endocarditis, which may also be explained in this case because *Gemella* is hemolytic [28]. His ESR was elevated, and although nonspecific, ESR is elevated in around 60% of cases of IE [4]. He also had low protein levels, which can indicate a systemic inflammatory response triggered by the infection, and an elevated BNP due to the strain on his heart. Although he had signs of infection like fever, his white blood count was not elevated, likely because his course was subacute at the time of evaluation. If no other causes for his condition were found after the workup or if he did not improve with antibiotic treatment, his primary care provider recommended a colonoscopy to rule out malignancy.

Because he experienced indolent dyspnea on exertion and leg swelling over the course of six weeks, he did not seek medical attention until his symptoms had worsened significantly, which in turn delayed treatment time. He did not have an audible murmur even though he had moderate to severe aortic regurgitation, and classic signs of IE such as immunological (Roth spots, Osler nodes) or vascular phenomena (Janeway lesions, conjunctival petechiae) were absent. He did not have some evident known risk factors for IE like a history of cardiac valve disease or IVDU, but a detailed history gave insight into the patient’s dental work, which is an important risk factor for his condition. Combining information from the history, physical exam, cultures, labs, and imaging helped the multidisciplinary team of clinicians to rule out other diagnoses quickly after the patient sought treatment, allowing for timely transfer of care so that surgical intervention could be performed as soon as possible.

## Conclusions

IE is an important diagnosis to keep in mind when a patient presents with nonspecific symptoms, even when clear risk factors may not be present. Early diagnosis using clinical tools such as labs and imaging, prompt initiation of tailored antibiotic therapy, and surgical intervention when indicated are important to improve patient outcomes in cases of IE. Upon reviewing the documented cases of *G. sanguinis* IE in the literature, it appears that valvular heart disease, often joined by other factors like poor dentition, has a strong association with the development of this infection. One significant limitation to diagnosis is the lack of understanding of why *Gemella* goes from a commensal organism to a pathologic one in those who are not otherwise immunocompromised. Future research is needed to learn more about the virulence mechanisms of *Gemella*, risk factors for *Gemella*-associated infections, and the generation of evidence-based guidelines for the management of *Gemella*-associated IE so that targeted therapies can be improved.
